# Aging Increases the Susceptivity of MSCs to Reactive Oxygen Species and Impairs Their Therapeutic Potency for Myocardial Infarction

**DOI:** 10.1371/journal.pone.0111850

**Published:** 2014-11-13

**Authors:** Liang Li, Yingfei Guo, Hongxia Zhai, Yaxin Yin, Jinjin Zhang, Haiwei Chen, Lei Wang, Na Li, Runmei Liu, Yunfeng Xia

**Affiliations:** 1 First Department of Cadres, First Hospital Affiliated to General Hospital of People's Liberation Army, Beijing, China; 2 Department of Emergency, First hospital affiliated to general hospital of People's Liberation Army, Beijing, China; University of Illinois at Chicago, United States of America

## Abstract

Myocardial infarction (MI) is one of the leading causes of death worldwide and Mesenchymal Stem Cells (MSCs) transplantation has been considered a promising therapy. Recently, it was reported that the therapeutic effectiveness of MSCs is dependent on the age of the donor, yet the underlying mechanism has not been thoroughly investigated. This study was designed to investigate whether this impaired therapeutic potency is caused by an increased susceptivity of MSCs from old donors to reactive oxygen species (ROS). The MSCs were isolated from the subcutaneous inguinal region of young (8–10 weeks) and old (18 months) Sprague–Dawley (SD) rats. By exposing these MSCs to H_2_O_2_, we found that the adhesion of MSCs from old donors was damaged more severely. Specifically, decreased expression of integrin and reduced phosphorylation of focal adhesion kinase Src and FAK were observed. Furthemore, H_2_O_2_ triggered an increased apoptosis of MSCs from old donors. To study the viability and therapeutic potency of MSCs from young and old donors *in vivo*, these MSCs were transplanted into acute MI model rats. We observed a more rapidly decreased survival rate of the old MSCs in the infarct region, which may be caused by their increased susceptivity to the micro-environmental ROS, as transplantation of the old MSCs with N-acetyl-L-cysteine (NAC), a ROS scavenger, protected them. The low viability of engrafted old MSCs consequently impaired their therapeutic effectiveness, judging by the histology and function of heart. Our study may help to understand the mechanism of MSCs-host interaction during MI, as well as shed light on the design of therapeutic strategy in clinic.

## Introduction

Ischemic heart disease, including myocardial infarction (MI) and heart failure happening after myocardial infarction, is the leading cause of death worldwide [1], with an increase of over 7 million patients a year [2]. Despite the great advance in surgery and medicine, over 50% of all patients succumb within 5 years of the initial diagnosis of heart failure, due to the inability of the traditional therapy in bringing the dead myocardium back to life and restoring the blocked small blood vessels [3]. In the last decade, stem cell based regenerative therapy has become a hot spot. Among various stem cells, Mesenchymal Stem Cells (MSCs) possess unique properties making them eligible for convenient and highly effective cell therapy, for they are: (1). Present in adult human body and have ethic advantage; (2). Multipotent to differentiate into numerous cells including cardiomyocytes, endothelial cells, etc.; (3) Easy to isolate and expand *in vitro* [4]. Recently, great successes have been achieved in transplanting MSCs to treat MI in different animal models including mouse [5], rat [6], swine [7] and canine [8], while many clinical trials [9]–[12] show significant ameliorated heart function of MI patients receiving MSCs transplantation. These evidences suggest MSCs transplantation a promising therapeutics for patients suffer MI.

One vital factor influencing the efficiency of MSCs transplantation therapy is the survival rate of engrafted MSCs. The infarction of myocardium generates a harsh micro-environment for engrafted MSCs [13]. Triggering by cell necrosis and inflammation, the concentration of Reactive Oxygen Species (ROS) may be increased over three-fold in the MI region [14]. High ROS were reported to induce apoptosis either directly [15] or through impairing cell adhesion, which leads to anoikis, the detachment induced apoptosis [16]. On the other hand, the property of engrafted MSCs is also crucial for their survival. Although MSCs have a considerable capacity of proliferation and differentiation, they surrender inevitably to the degeneration caused by aging, like the entire human body. With aging, telomere shortening [17], accumulation of senescent markers as β-galactosidase and the impairment of proliferation [18] were found in MSCs *in vitro*. MSCs isolated from 56 week-old Wistar rats showed accumulated levels of oxidized proteins and lipid, increased apoptosis and reduced proliferation, as well as impaired potential for differentiations compared with that isolated from 8–12 week-old rat [19]. Recently, Khan. M. et. al [20] reported a functional incapacitation of aged MSCs for the treatment of myocardial infarction. Thus, are MSCs from an old donor more susceptible to ROS? What is the mechanism and to what extent is the therapeutic function of aged MSCs impaired? These are crucial questions because 80% of patients with ischemic heart disease were older than 65 years [21], and MSC-based therapy in clinic is now mainly in an autologous way.

In this present study, we isolated MSCs from 8–10 week-old and 18 month-old male Sprague–Dawley rats, namely young and old MSCs. *In vitro* studies showed that MSCs from old donors were more susceptible to exogenous ROS induced adhesion impairment and apoptosis. By transplanting young and old MSCs to treat the MI model rats, we found a more rapidly decreased survival rate of the old MSCs engrafted as time went by, compared to that of the young MSCs. However, when co-injecting old MSCs with NAC, a ROS scavenger, a similar number of the remained old and young MSCs were observed. Using echocardiography and hemodynamics examinations to evaluate heart function 4 weeks after transplantation, we observed significantly different therapeutic effectiveness between young and old MSCs. Our study suggested that aging increases the susceptivity of MSCs to reactive oxygen species and thus impairs their therapeutic potency for myocardial infarction.

## Materials and Methods

All animal experiment procedures in this study were conducted in compliance with the Guide for the Care and Use of Laboratory Animals (NIH publication no. 85-23, revised 1996) and were approved by the Institutional Animal Care and Use Committee (IACUC) of General Hospital of PLA.

### MSCs isolation and culture

MSCs were isolated from the subcutaneous inguinal region of 8–10 weeks or 18 months old male Sprague–Dawley (SD) rats following protocol previously described [22]. In brief, the adipose tissue was digested with 0.075% type I collagenase (Sigma) in a 5% CO_2_ incubator for 1 h at 37°C. This mixture was filtered through a 140 µm nylon mesh and centrifuged at 1.200×g for 10 min at 4°C. The pellet was washed and loaded onto a Percoll density gradient (1.077 g/ml) and finally centrifuged at 1.000×g for 20 min at 4°C. The resulting interphase was washed and cultured in Dulbecco's modified Eagle medium-low glucose (DMEM-LG; Gibco, Grand Island, NY) supplemented with 10% fetal bovine serum (MDgenics, St. Louis, MO) in a 5% CO2 incubator at 37°C for four to five passages expansion before transplantation. The early passage (passage 3) MSCs were characterized by flow cytometry and analyzed for their multi-directional differentiation ability.

### Flow cytometry

For flow cytometry analysis, MSCs were washed with phosphate buffered solution (pH 7.4) and incubated in the dark for 1 h at room temperature with CD90-FITC, CD29-FITC, CD45-FITC or CD34-FITC (BD Pharmingen, San Diego, CA, USA) antibodies. The specific fluorescence of 10,000 cells was analysed on FACScalibur (Becton Dickinson, Franklin Lakes, NJ, USA) using Cell Quest Pro software.

### Multi-directional differentiation

Differentiation of MSCs was induced according to established protocols [23]. For adipocytes differentiation, MSCs were cultured in adipocyte differentiation medium containing 0.5 mM isobutylmethylxanthin, 60 mM indomethacin, 100 nM dexamethasone and 10 mg/ml insulin. The presence of adipocytes was verified by staining for triglycerides with Oil Red O, which is an indicator of intracellular lipid accumulation. For osteogenic differentiation, MSCs were cultured in osteogenic differentiation medium containing 10% FBS, 10 nM dexamethasone, 100 mM L-ascorbic acid, and 10 mM β-glycerophosphate. To indicate active osteoblasts, these cultures were stained with Alizarin Red S to identify calcium deposition.

### Rat MI model

Rat myocardial infarction model were established following protocol previously reported [16]. In brief, 8–10 week-old Sprague-Dawley male rats (about 250 g) were anaesthetized with sodium pentobarbital (40 mg/kg). After exposing the heart by left side limited thoracotomy, the left anterior descending coronary artery was ligated 3 mm below its origin with a 6-0 silk suture. Ischemia was confirmed by the blanching of the myocardium and dyskinesis of the ischemic region, and restoration of normal rubor indicated successful reperfusion. These MI model rats were randomly assigned into 3 groups for MSCs transplantation.

### Labeling of MSCs and transplantation

MSCs from old and young donor were labeled with DiI (1,1′-dioctadecyl-3,3,3′3′-testramethylindocarbocyanine perchlorate; Sigma) as previously reported [24]. Labeled MSCs were then trypsinized and suspended to a concentration of 2×10^7^ cells/ml in PBS. For transplantation, after surgical occlusion of the left anterior descending coronary artery, 2×10^6^ young or old MSCs in 100 µl PBS, 2×10^6^ old MSCs with 1 mM NAC (N-acetyl-L-cysteine; Sigma) in 100 µl PBS, or 100 µl PBS only were injected intramyocardially at three sites in the border of the infarct area, respectively. The 4 groups of MI rats with transplantation, namely (1) PBS control group (n = 10), (2) Old MSCs transplantation group (n = 20), (3) Young MSCs transplantation group (n = 20), (4) Old MSCs + NAC transplantation group (n = 20), were used in this study.

### Assessment of engrafted MSCs viability and heart histology

At day 0 and day 7, five rats each from the above 4 groups were sacrificed. The hearts were removed and cryo-sections (5 µm) were made. Viable DiI-labeled MSCs that were transplanted were observed and counted by fluorescence microscopy at four separate fields.

To measure infarct size, the remaining 10 rats of each group were sacrificed 4 week after MSCs transplantation. The hearts were embedded in paraffin and sections were cut from the mid-left ventricle and base. All sections were stained with Masson's Trichrome. Fibrosis and total left ventricle area of each image were measured using the Image Pro Plus. The percentage of the fibrotic area was calculated as (fibrosis area/LV area)×100. All histological data in this study was treated and analyzed by three persons blinded.

Also, to measure capillary density in the infarcted myocardium, Immunohistochemistry was performed. The paraffin sections was stained with mouse monoclonal anti-CD31 (Cymbus Biotechnology, Inc.). For quantification, capillaries were counted in ten randomly chosen fields at 20× and the mean number of capillaries per field was used for statistical analysis.

### Assessment of heart function

4 week after MSCs transplantation, the heart function of MI model rats were evaluated by echocardiography and hemodynamics examination. Being anaesthetized with sodium pentobarbital (40 mg/kg) by intraperitoneal injection, the rats were first examination for echocardiography by Sequoia 512 Color Ultrasound System (Siemens). The left ventricular ejection fraction (LVEF) and left ventricular fractional shortening (LVFS) were measured. Then, hemodynamics examination was performed. The right carotid artery was cannulated with a micro tip pressure transducer catheter (SPR-839, Millar Instruments, Inc., Houston, TX, USA) connected to MPCU-400 P-V signal conditioning hardware for data acquisition. Haemodynamic parameters and PV loops were recorded and Left ventricular end diastolic pressure (LVEDP), Pmax and dp/dtmax were evaluated using Millar's PVAN 3.0 software.

### Immunoblot analysis

Young and old MSCs with or without 50 uM H_2_O_2_ treatments for 1 hour were washed twice in cold PBS and lysed in buffer containing 20 mM Tris (pH 7.5), 150 mM NaCl, 1 mM Na2-EDTA, 1 mM EGTA, 1% Triton, 2.5 mM sodium pyrophosphate, 1 mM b-glycerophosphate, 1 mM Na3VO4, 1 mg/ml of leupeptin, and 1 mM phenylmethylsulfonyl fluoride. Protein concentrations were determined using the Bradford Protein Assay Kit (Bio-Rad). Proteins were separated in a 10% sodium dodecyl sulfate–polyacrylamide gel and transferred to a polyvinylidene difluoride membrane (Chemicon International Inc.). After blocking with TBS based 5% nonfat milk containing 0.1% Tween 20 for 1 hour at room temperature, the membrane was washed twice with TBS-T and incubated with primary antibody overnight at 4°C. After washing, the membrane was incubated for 1 hour at room temperature with horseradish peroxidase-conjugated secondary antibodies. Bands were detected by enhanced chemiluminescence reagent (Santa Cruz Biotechnology). Quantification of band intensities was made using the Photo-Image System (GE life sciences).

### Assays for MSCs adhesion

Suspensions of 2×10^4^ viable young and old MSCs were plated to each well of four-well plates with or without the presents of 25, 50, and 100 µM H_2_O_2_ as an exogenous ROS source. These cells were allowed to attach for 1 hour at 37°C in 5% CO_2_. Then plates were carefully washed three times with PBS, and four separate fields were photographed with a phase-contrast microscope. The adhered cells were counted blinded. Each experiment was performed in triplicate wells and repeated at least three times.

### Reverse Transcription PCR Analysis

The expression levels of the integrin β1 (also termed CD29) and αv genes of young and old MSCs treated with 50 µM H_2_O_2_ (or PBS as control) were analyzed by reverse transcription polymerase chain reaction (RT-PCR). Total RNA was extracted with Trizol reagent (Invitrogen). 2 µg RNA were reverse-transcribed into cDNA by random primers using Technical Bulletin Reverse Transcription System (Promega). Real-time PCR analysis was performed on Corbett 6200 using SYBR Green PCR mix (TOYOBO Co.). Ct values of GAPDH (as internal control) were subtracted from Ct values of the genes of interest (ΔCt). ΔCt values of genes from young MSCs treated with PBS were set as 100% and compared by other groups. The primers for integrin β1 and αv were purchased from Qiagen (Quanti-Tect Primer Assays). The Primers for GAPDH were synthesized by Invitrogen using the following sequence (Sense; Antisense): 5′-CTCCCAACGTGTCTGTTGTG-3′ and 5′- TGAGCTTGACAAAGTGGTCG-3′.

### Apoptosis assay

To measure apoptosis of MSCs exposed to exogenous H_2_O_2_, young and aged MSCs were treated with 25 µM, 50 µM or 100 µM H_2_O_2_ for 24 h. Thereafter, these cells were trypsined and stained with Anexin V apoptosis kit (Sigma), then analyzed on FACScalibur (Becton Dickinson) using Cell Quest Pro software.

### Immunofluorescence

For immunofluorescent staining, young and old MSCs were plated on coverslips. After exposing to 50 µM H_2_O_2_ (or PBS as control) for 1 h, the cells were washed with PBS and fixed in 4% paraformaldehyde/phosphate-buffered saline. Being permeabilized with 0.2% Triton X-100/phosphate-buffered saline, the cells on coverslips were incubated with mouse anti-integrin β1 antibody, followed by incubation with Alexa Fluor 555 conjugated Donkey anti-Mouse IgG (Invitrogen, Inc.). The cell nucleus was stained by DAPI. Samples were examined and photoed with a confocal laser scanning microscope (Olympus, Tokyo, Japan).

### Statistical Analysis

All quantified data represent an average of at least triplicate samples. The error bars represent the standard deviation of the mean. Statistical significance was analyzed by Student's t-test using Graphpad Prism 5.0 software. A p value <0.05 was considered significant.

## Results

### Characterization of MSCs from young and aged donor rats

**Figure 1:**
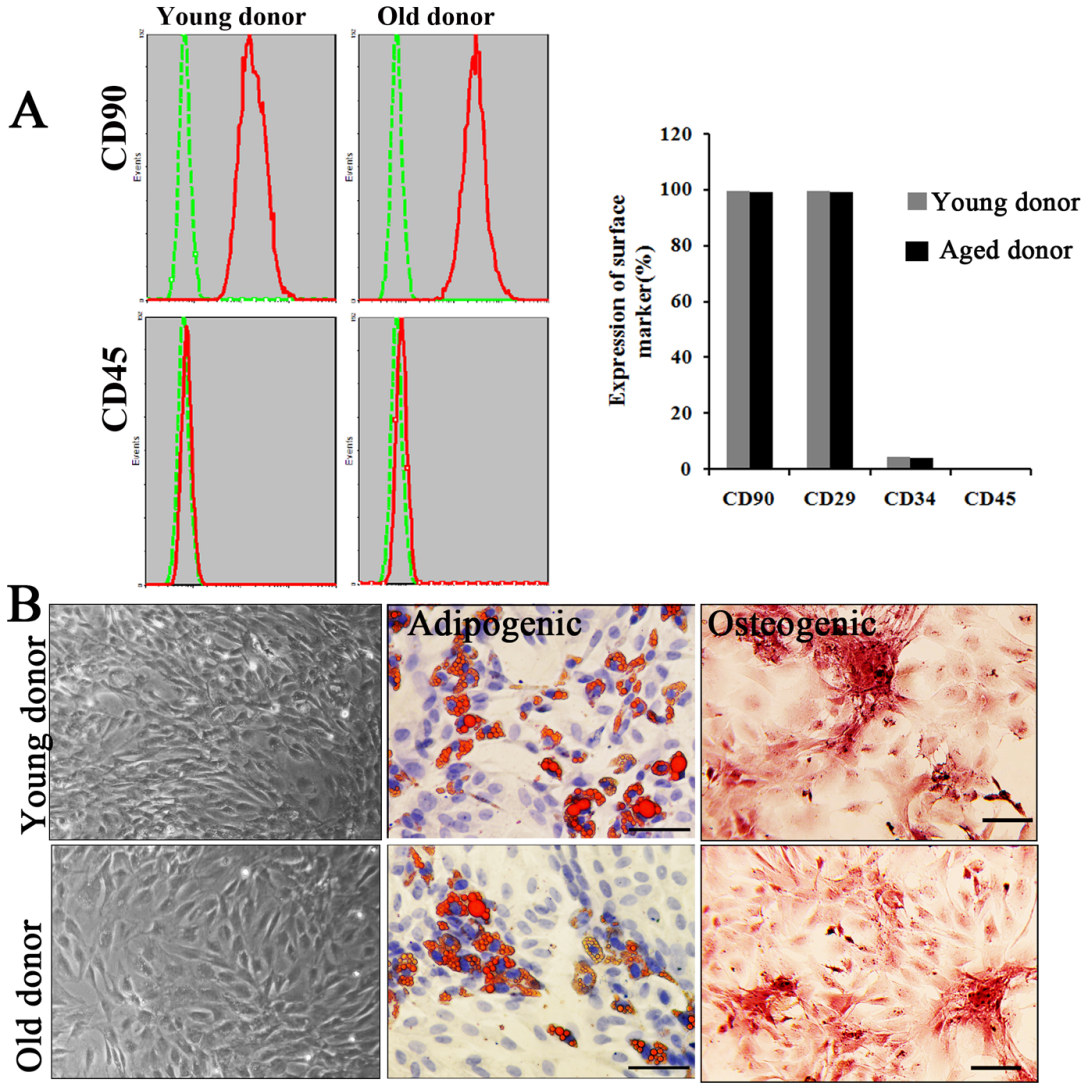
Characterization of MSCs from young and old donors. A. Flow cytometry analysis of cultured MSCs. Both MSCs from young and old donor were positive for CD90, CD29 and negative for haematopoietic markers CD45FITC and CD34PE. B. Morphology and multiplex differentiation potential of cultured MSCs. The presence of adipocytes was verified by staining for triglycerides (red) with Oil Red O and the presence of active osteoblasts were verified by staining for calcium deposition (red) with Alizarin Red S. Representative images were shown. Scale bar: 100 µm.

Mesenchymal stem cells isolated from young and old donor rats were first characterized using flow cytometry for their expression of MSCs markers. As shown in Figure 1A, most of the young and old MSCs we isolated were positive for CD90 and CD29 expression (>99%), and negative for haematopoietic markers CD34 and CD45. For further confirmation, the MSCs were examined for their multiplex differentiation potential. By changing of culture conditions, these MSCs could differentiate into adipocytes and osteogenic cells, respectively (Figure 1B). No significant differences, neither in MSCs markers' expression nor in differentiation potential, were found between MSCs from young and old donors.

### MSCs from old donors are more susceptible to exogenous ROS induced adhesion impairment and apoptosis

**Figure 2:**
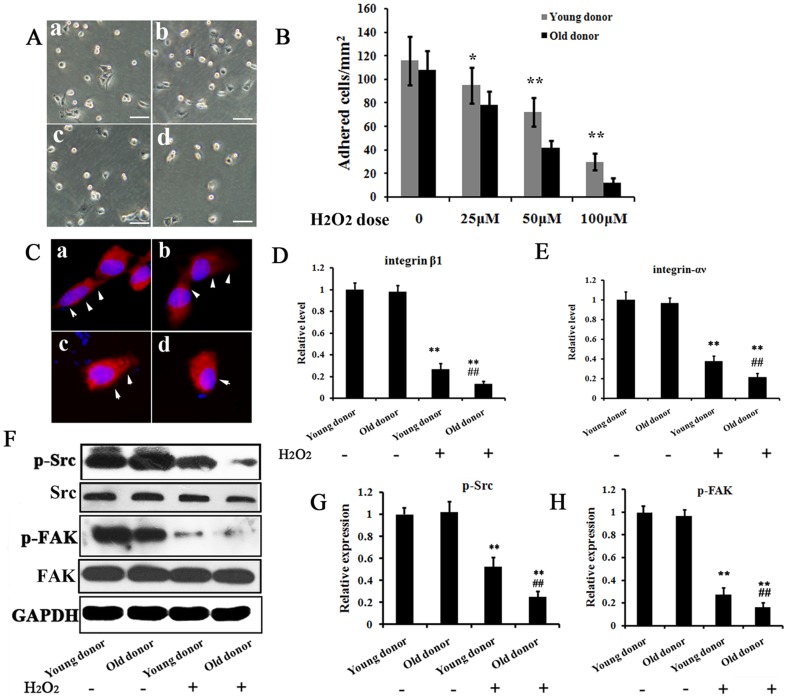
Susceptibility of MSCs from young and old donors to ROS. A. Representative images of adhered cells. ‘a’ and ‘b’: Adhesion of MSCs from young and old donors without H_2_O_2;_ c′ and ‘d’: Adhesion of MSCs from young and old donors under 50 µM H_2_O_2_. Bar = 100 µm. B. H_2_O_2_ influences the MSC adhesion in a dose-dependent manner. * P<0.05, **P<0.01. C. Immunofluorescence stain for expression of integrin β1 (red) in MSCs of above adhesion assay. Cell nucleus was stained with DAPI (blue). Arrows indicate the spread morphology of MSCs. Arrows indicate the spread morphology of cells. D and E. The relative expression level of integrin β1 and αV were analyzed by RT-PCR in MSCs from young and old donors with indicated treatment. The mRNA level of PBS treated MSCs from young donors was set as 100%. F-G. Phosphorylation of Src and FAK were analyzed by west-blot in MSCs from young and old donors with indicated treatment. The phosphorylation level of PBS treated young MSCs was set as 100%. ** p<0.01 compared with young and old MSCs without H_2_O_2_ treatment; ##** p<0.01 compared with young MSC with H_2_O_2_ treatment.

ROS impairs adhesion of stem cells [16]. To test whether old MSCs were more susceptible to ROS, adhesion assays were performed with MSCs from young and old donors by treating them exogenously with H_2_O_2_. As shown in Figure 2A and 2B, H_2_O_2_ impaired the adhesion of MSCs in a dose dependent manner. Meanwhile, a greater decrease in the adhesion of the old MSCs, compared with that of the young MSCs at each H_2_O_2_ concentration, was observed. In addition, we evaluated the altered expressions of adhesion-related integrin molecules in H_2_O_2_-treated MSCs using immune-fluorescence (Figure 2C) and RT-PCR (Figure 2D and 2E). A significant change in cell morphology and down-regulation of integrin β1 (also termed CD29) and αV were observed in H_2_O_2_-treated old MSCs, compared with H_2_O_2_-treated young MSCs and non-treated MSCs. Furthermore, we examined the phosphorylation of kinases FAK and Src, as they play vital roles in cellular focal adhesion by regulating the actin cytoskeleton. As seen in Figure 2F-2H, H_2_O_2_ treatment significantly reduced the phosphorylation of both kinases, with old MSCs suffered more than the young ones.

**Figure 3:**
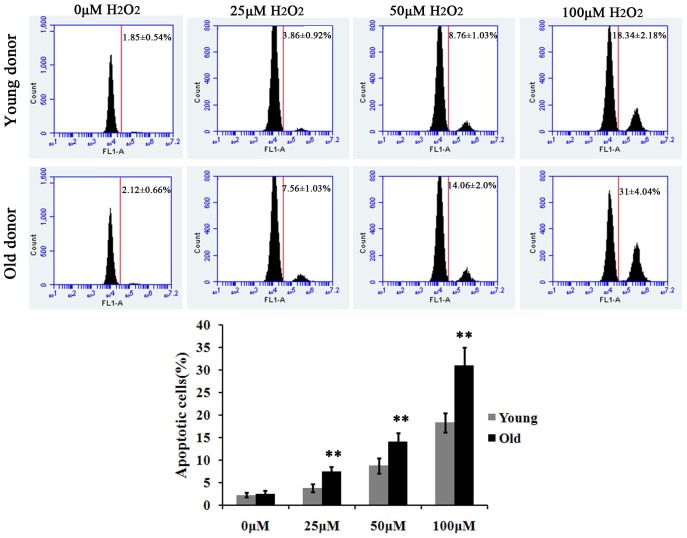
Apoptosis of MSCs from young and old donors induced by ROS. MSCs from young and old donors were treated by 0, 25, 50 or 100 µM H_2_O_2_ for 24 h. Then Flow cytometry analysis was performed to detect cell apoptosis using Anexin V apoptosis kit. ** p<0.01

We next examined the viability of young and old MSCs in ROS environment. As shown in Figure 3, the apoptosis of MSCs were triggered at an H_2_O_2_ concentration of 25 µM and gradually increased in a dose-dependent manner. A two-fold increase in apoptosis of old MSCs was observed compared with that of the young MSCs at a same H_2_O_2_ concentration. Together, these *in vitro* data suggest that MSCs from old donors are more susceptible to exogenous ROS induced adhesion impairment and apoptosis than the ones from young donors.

### Lower viability of MSCs from old donors in myocardial infarction region after transplantation

**Figure 4:**
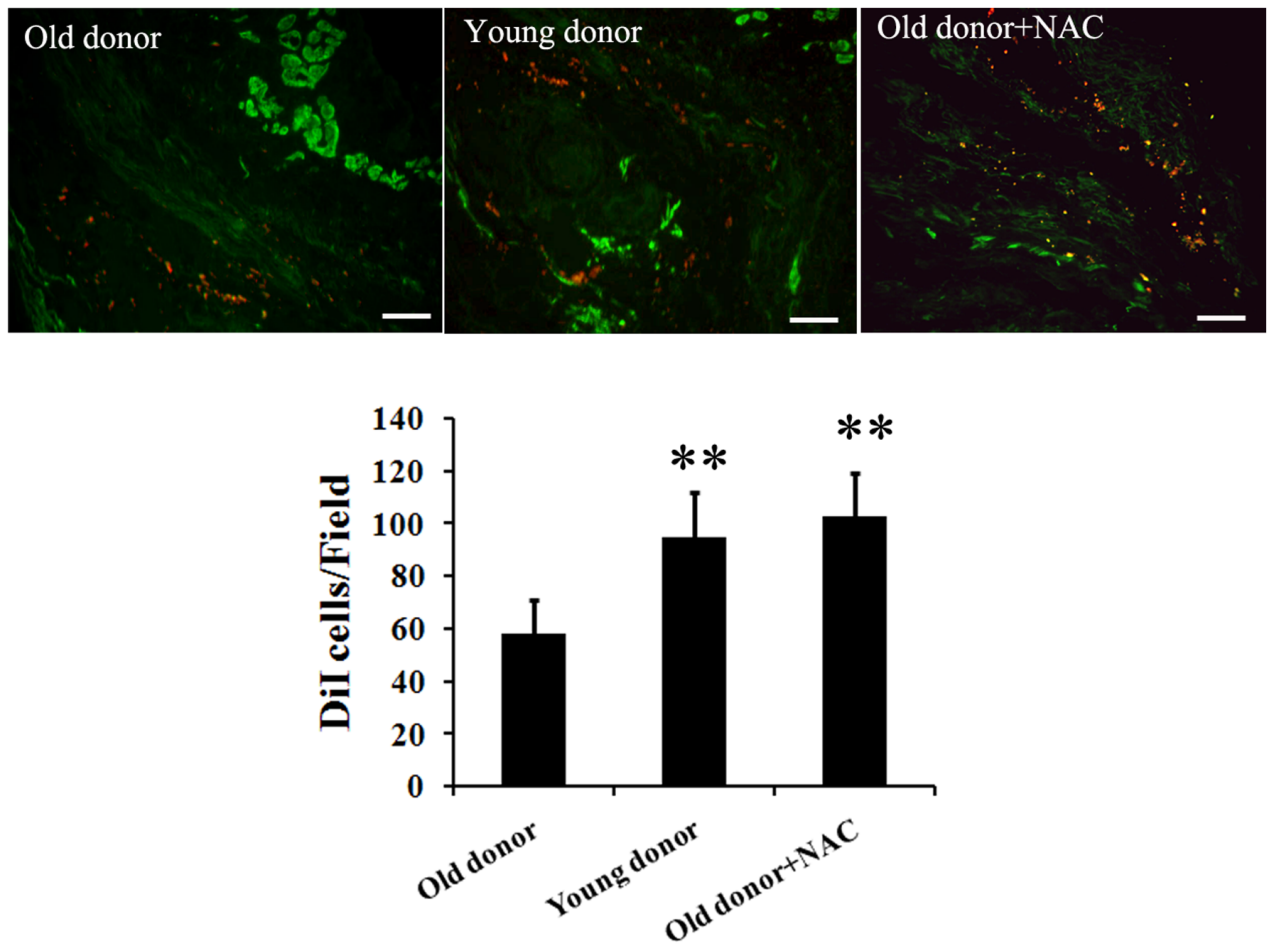
Viability of engrafted MSCs from young and old donors at day 7 after transplantation. MSCs were pre-stained with DiI before transplantation. At Day 7 after transplantation, MSCs number in MI region of each group was counted under fluorescence microscopy. Representative images and statistics were shown. N = 5 per group. Scale bar: 100 µm. ** p<0.01 all compared with old donor group.

To investigate whether aging affects the implantation and viability of MSCs *in vivo*, an acute myocardial infarction rat model was implicated. It has been reported that the concentration of ROS may be increased over three-fold in the MI region [14]. Consistently, we found a significantly elevated ROS level in cardiac tissue of the MI model rats by measurement of malondialdehyde (MDA) as previously reported [25] (data not shown). DiI-stained MSCs from young and old donors were transplanted in the border of the infarct region of the MI model rats. At day 0 and day 7 after transplantation, the hearts were sectioned and the fate of the engrafted MSCs was investigated using confocal microscopy. At day 0, right after transplantation, a similar number of young and old MSCs were observed (data not shown). However, as shown in Figure 4, one week after transplantation, fewer old MSCs remained, whose number is only about half of that of the young MSCs. In order to study whether this lowered viability of MSCs from old donors was caused by the increased susceptibility to environmental ROS, we also included a positive control group, where a same number of old MSCs were transplanted in combination with the ROS scavenger NAC. Interestingly, the number of NAC treated old MSCs remained was significantly larger than that of old MSCs injected alone, whereas no significant difference was found between the number of remained young MSCs and the NAC treated old MSCs. Together, these data indicate that old MSCs engrafted are less resistance to the ROS in myocardial infarction micro-environment than the young ones.

### Impaired potential of old MSCs in repairing infarcted myocardium

**Figure 5:**
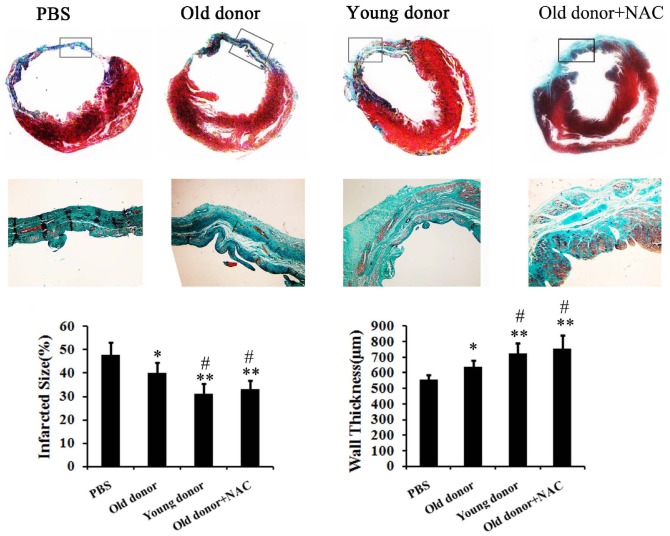
Histology of heart of MI model rats transplanted with young or old MSCs. The hearts of MI model rats were sectioned and stained with Masson's Trichrome 4 weeks after transplantation. Fibrosis and total left ventricle area were measured. The percentage of the fibrotic area was calculated as (fibrosis area/LV area) ×100%. N = 10 per group. * p<0.05 compared with PBS group; ** p<0.01 compared with PBS group; # p<0.05 compared with Old donor group; ## p<0.01 compared with Old donor group.

As the viability of young and old MSCs differed, will there be a significant difference in their potential in repairing infarcted myocardium? To answer that, 4 weeks after transplantation, the infarct size and wall thickness of hearts were measured. Compared with PBS control group, both transplantation of young and old MSCs reduced the infarct size and increased the wall thickness. However, when compared with young MSCs, significant defect of old MSCs was observed (Figure 5).

**Figure 6:**
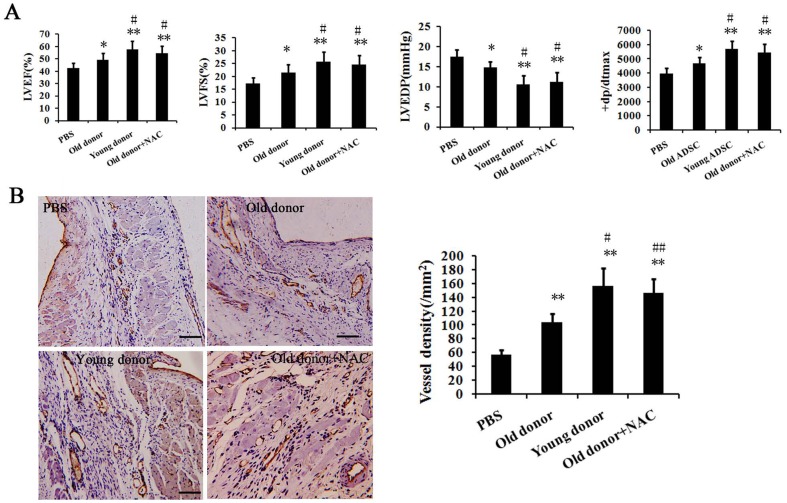
Assessment of heart function of MI model rats transplanted with young or old MSCs. A. The heart functions of MI model rats were evaluated by echocardiography and hemodynamics examination. LVEF: Left ventricular ejection fraction; LVFS: Left ventricular fractional shortening; LVEDP: Left ventricular end diastolic pressure. B. Capillary density in the infarcted myocardium was measured by Immunohistochemistry. Capillaries were indicated by CD31 stain. Representative images and statistics were shown. Scale bar: 100 µm. * p<0.05 compared with PBS group; ** p<0.01 compared with PBS group; # p<0.05 compared with Old donor group; ## p<0.01 compared with Old donor group.

Furthermore, we assessed the heart function using echocardiography and hemodynamics examinations. As shown in Figure 6A, parameters including the left ventricular ejection fraction (LVEF), left ventricular fractional shortening (LVFS), left ventricular end diastolic pressure (LVEDP) and dp/dtmax suggested a significant amelioration after young MSCs' transplantation, while transplantation with old MSCs performed not so well as that with the young ones. In addition, capillary density in the infarcted myocardium was measured by Immunohistochemistry. As shown in Figure 6B, the mean micro-vessel count per field was significantly higher in the young MSCs' transplantation group than the group transplanted with old MSCs. Notably, seen from the results of both the heart histology and the heart function, the transplantation of old MSCs together with NAC had a similar therapeutic efficiency as the transplantation of young MSCs alone. Taken together, these data suggest an impaired potential of aged MSCs in repairing infarcted myocardium, which is caused largely by the ROS in MI micro-environment.

## Discussion

In the last decade, MSCs-based cell therapy has been shown great solicitude for, with great successes been achieved in transplanting MSCs to treat MI in animal models as well as in clinic trials. Previously, lower efficacy of old MSCs than the young ones in myocardial repair has been confirmed by independent studies [20], [26] and furthermore different potential mechanisms have been proposed, such as deteriorated paracrine capacity and impared angiogenic capacity. However, the causes why the efficacy of MSCs on myocardial repair after ischemia was attenuated with aging were far from thoroughly demonstrated. In the current study, our purpose is to determine whether other causes existed in addition to the previous findings that aging influenced the therapeutiac efficacies of MSCs. We show that aging increases the susceptivity of MSCs to ROS and impairs their therapeutic potency for myocardial infarction. To our knowledge, this is the first evidence that MSCs from old donors were more susceptible to ROS induced adhesion impairment and apoptosis, leading to a more rapidly decreased survival rate, and thus resulting in a dampened therapeutic effectiveness. Our study will provide supplementary knowledge to the previous reports why old MSCs are less effective than the young ones after myocardial transplantation.

The number of hospitalizations of post-ischemia heart failure, as either the principal or the secondary diagnosis in patients over the age of 65, has increased by 70% to 100% over the last quarter century [27]. MSCs-based cell therapy therefore has been considered a breakthrough strategy for its potential in regenerating cardiomyocytes and restoring heart function. Back to 2001, two landmark studies of Orlic et al. [28], [29] showed transplantation of bone marrow cells could generate de novo myocardium. Thereafter, MSCs transplantation was carried out by several clinic trials, and a promising therapeutic potential was reported. For example, Chen et al. [9] (a controlled clinic trail of 69 acute MI patients in 2004), Ruan et al. [10] (20 acute MI patients in 2005) and Katritsis et al. [11] (11 acute MI patients in 2005) all reported significant improvements of heart function after autologous MSCs transplantation. In 2006, a double-blind, placebo-controlled multicentre trial involving 204 patients was reported by Schachinger et al. [12], showing that after 1 year follow up, the combined endpoint death, recurrence of myocardial infarction, and rehospitalization for heart failure were significantly reduced in patients receiving bone-marrow-derived stem cells transplantation (P = 0.006).

However, as autologous MSCs transplantation was favoured in clinic, and most patients were over 60 years old, one question arises – Are MSCs from old donors qualified to do the job? Although with a profound potential of proliferation and differentiation, MSCs surrender to aging as well [30]. As reported by Stolzing et al. [19], MSCs isolated from 56 week-old Wistar rats showed increased levels of apoptosis and reduced proliferation than that isolated from 8–12 week-old rat. Recently, Khan et al. [20] reported an increased expression of cellular senescence marker and a decreased paracrine of growth factors in MSCs from aged animals, while transplantation with MSCs from young donor exhibited a more significant improvement in heart function of MI model mice than with aged MSCs. In consistent, we found an impaired therapeutic efficiency of transplantation using MSCs from old donors. Furthermore, our data suggest that this impairment may be caused directly by a significantly decreased viability of old MSCs engrafted, in which the micro-environmental ROS in the MI region may play important roles. In 2010, Song et al. reported that the co-injection of MSCs with the free radical scavenger, NAC (N-acetyl-L-cysteine) protected MSCs from ROS and enhanced their therapeutic efficiency [16]. In our study, in order to investigate whether MSCs from old donors were more vulnerable to the micro-environmental ROS in the MI region *in vivo*, besides the old and young MSCs transplantation groups, we introduced a group in which 1 mM NAC was co-injected with the old MSCs. Interestingly, we found a similar number of NAC treated old MSCs and young MSCs remained one week after transplantation, whereas the number of survived MSCs from old donors was only about a half of that of survived MSCs from young donors. In addition, judging by the histology and function of heart, we found an impaired therapeutic efficiency transplanting MSCs from old donors. Since the NAC plays its role as a ROS scavenger but does not have a significant therapeutic effect in treating MI without MSCs [16], we may safely indicated that MSCs from old donors has lower viability in vivo in the MI region due to their increased susceptivity to the environmental ROS. Therefore, even the ability of differentiation and paracrine equal between MSCs from young and old donors, there is no enough MSCs to play their roles in the case of old MSCs' engrafting.

Of note, in this study, we showed that that there was no significant difference between old MSCs and young MSCs for their multi-lineage differentiation (Figure 1B) and adhesion ability (Figure 2A and B) without H_2_O_2_ stress, which seems not consistent with other previous reports. Actually, we found that deteriorated functions of MSCs with aging (e.g. capability of self-renewal, proliferation and differentiation) were more significant when the cells were passaged more times *in vitro*. In early passages (no more than three passages), the difference between young-donor-derived MSCs and old-donor-derived ones was not so significant, but it was significant when the cells were under oxidative stress. To confirm that the elder MSCs were more susceptible to ROS, we used early passaged MSCs(P2–P3) in the study so that the functions between young MSCs and old one were comparable under normal condition. In this way, it should be clearer that the differences between young MSCs and old one under ROS condition were resulted from their different accommodative capacities against ROS, that the capacity of old MSCs deteriorated or old MSCs were more susceptible to ROS.

To survive, cells require an adequate interaction between them and the extracellular matrix, otherwise they will undergo apoptosis, known as anoikis. Thus, the viability of engrafted MSCs also depends on cell adhesion. However, the infarction of myocardium created a harsh micro-environment, including an accumulation of ROS, which has been reported to hinder cell adhesion. Therefore, we postulated that the low survival rate of MSCs from old donor may be caused by an enhanced susceptibility to environmental ROS. By adhesion assay and apoptosis assay, we found that ROS caused more damage in the adhesion of old MSCs than of the young ones, which further increase the old MSCs' apoptosis indirectly. Specifically, we tested the expression and function of integrin β1 and αV, for they were reported to play a key role in mediating cells adhesion to the extracellular matrix and regulating intracellular signaling pathway that control cytoskeletal organization, force generation and survival. Interestingly, a significant decreased expression of integrin β1 and αV, as well as a reduced phosphorylation of focal adhesion kinase including Src and FAK, were found in ROS treated old MSCs. This was consistent with the previous report that ROS inhibits adhesion of MSCs implanted into Ischemic myocardium via interference of focal adhesion complex [16]. However, the underlying mechanisms of increased susceptibility of old MSCs to ROS may be rather complicated, which may include disrupted antioxidant system or anti-aging system, alterred apoptosis signaling, deteriorated function of organelles as well as attenuated adaptive capacities, ect. In this study, our main purpose is to provide the evidence that MSCs from old donor are more susceptible to ROS. Nevertheless, further studies were needed and our focus will be on the adaptive capacities of MSCs with aging against adverse environment, e.g. microRNAs, which are important regulators for cell functions.

Moreover, in our current study, we transplanted MSCs from young and aged donor to 8–10 week-old MI model rats. Actually, the age of the host may influence the outcome too, as Fan et al. [31] reported recently that aging might augment ROS formation and affect reactive nitrogen species (RNS) level after myocardial ischemia/reperfusion in both humans and rats. Therefore, using MSCs from old donor to treat old patients may make things even worse, indicating autologous transplantation a bad choice among aged patients. Thus, what can be done to better the effectiveness of MSCs transplantation for aged patients in clinic? Based on this study, our suggestions were as follows. First, for those who are old or feeble, their MSCs' viability and function should be assessed. According to the result, a change of the autologous transplantation to an allogeneic one would be recommended. Second, since enhanced ROS in the micro-environment could impair the engrafted MSCs, a co-treatment of transplantation with free radical scavengers should be tested by clinic trials. Actually, N-acetyl-L-cysteine, a free radical scavenger, was reported to enhance the effectiveness of MSCs transplantation in an MI rat model [16]. Furthermore, pretreating MSCs with growth factors, reconditioning MSCs with hypoxia, or even genetically modification of MSCs may also help to improve MSCs transplantation therapy, as indicated by several animal experiments [32]–[34]. Nevertheless, further studies should be done to evaluate the safety and effectiveness of these methods.

In this study, we tracked engrafted MSCs *in vivo* for their viability by pre-labeling them with DiI. For tracking engrafted cells in vivo, several methods have been reported. Generally, they may be categorized into three strategies: 1)transplanting cells into gender- or species-mismatched recipients, then the cells were detected based on the gender- or species-specific markers, such as detecting male donor cells in female recipients based on Y chromosome, or detecting human cells in animal recipients based on the human-specific antibodies; 2) labeling cells with marker genes, such as GFP, LacZ, Fluc, ect., then the cells were detected based on these genes; 3) labeling cells with fluorescent dyes, such as DAPI, DiI ect, then the cells were detected on these dyes. In the study, we performed stem cells transplantation in rat models of MI. It was generally considered that female rats possessed potent self- recovery capability after MI [35]. Therefore, male rats were usually used as MI models. In addition, to minimize the potential immune rejection, the cell donors and recipients are syngeneic in our study. Actually, we have also tried to label MSCs with GFP viral vectors. However, we found that viral transduction would significantly influence the functions of old-donor derived MSCs. Collectively, we used DiI-labeling to analyze MSCs viability in the study.

To date, MSCs from various origins have been tested in order to treat MI, including MSCs derived from bone marrow, adipose tissue, placenta, amnion and umbilical cord. The bone marrow derived MSCs (BM-MSCs) have been widely used, while adipose tissue derived MSCs (AT-MSCs) have been considered as an alternative, because liposuction is less invasive than bone marrow aspiration and can be easily and reproducibly replicated in a large-scale. Although there are differences between these two kinds of MSCs, such as variation in cell surface markers, and tendency of differentiation, no significant difference has been reported in their therapeutic mechanism and functions in treating MI. The discovery that MSCs functions, such as capability of self-renewal, proliferation and differentiation, start to deteriorate as they get older was first made in BM-MSCs [17]–[19]. Khan. M. et.al [20] reported a functional incapacitation of aged BM-MSCs for the treatment of myocardial infarction. In consistent with their finding, we here showed the impaired therapeutic efficiency of transplantation using AT-MSCs from old donors, and suggest that this impairment may be caused directly by a significantly decreased viability of old MSCs engrafted, which resulted from ROS. There is also report that compared morphological, molecular and functional differences of adult bone marrow- and adipose-derived stem cells isolated from rats of different ages, which demonstrated that both BM- and AT-MSCs from aged animals expressed similar markers of senescence [36]. In addition, more recently, Wu et al. reported that donor age negatively affects the immunoregulatory properties of both adipose and bone marrow derived MSCs [37]. Therefore, though the increased susceptibility of old MSCs were observed with adipise-derived MSCs, we could infer from the previous studies that the phenomenon may be universal, not just for adpose source. Of course, this needs further confirmation in future.

In summary, we show that MSCs from old donors were more susceptible to ROS induced adhesion impairment and apoptosis. The survival rate of MSCs from old donors decreased more rapidly in MI region after transplantation, thus dampening their therapeutic effectiveness. Therefore, our study may help to understand the mechanism of MSCs-host interaction during MI, as well as shed light on the design of therapeutic strategy in clinic.
